# Clinico-Microbiological Profile of Carbapenem-Resistant Acinetobacter baumannii Isolates From Intensive Care Unit Patients and In Vitro Activity of Sulbactam-Durlobactam: A Cross-Sectional Study From Eastern India

**DOI:** 10.7759/cureus.104172

**Published:** 2026-02-24

**Authors:** A. Susanna, Nipa Singh, Gaurav Verma, Ipsa Mohapatra, Sujit Pradhan, Subhra Snigdha Panda, B. Prince, Dipti Pattnaik, Pragyan Parimita Mishra

**Affiliations:** 1 Department of Microbiology, Kalinga Institute of Medical Sciences, Bhubaneswar, IND; 2 Department of Community Medicine, Kalinga Institute of Medical Sciences, Bhubaneswar, IND; 3 Department of Critical Care Medicine, Kalinga Institute of Medical Sciences, Bhubaneswar, IND; 4 Department of Nursing, Kalinga Institute of Medical Sciences, Bhubaneswar, IND

**Keywords:** antimicrobial resistance, carbapenem-resistant acinetobacter baumannii, icu infections, multidrug-resistant pathogen, sulbactam-durlobactam

## Abstract

Background

Carbapenem-resistant *Acinetobacter baumannii* (CRAB) causes severe infections in intensive care units (ICUs), with increased morbidity and mortality. The pathogen exhibits extensive antimicrobial resistance, substantially limiting available therapeutic options. Recently, the emergence of sulbactam-durlobactam, a novel β-lactam/β-lactamase inhibitor combination, has renewed interest as a potential treatment option. The study evaluates the clinical and microbiological profile of CRAB isolates, assesses the in vitro efficacy of sulbactam-durlobactam in these isolates, and analyzes key risk factors and clinical outcomes in patients developing CRAB infection.

Methodology

This cross-sectional study was conducted over a period of six months (July-December 2024) at a tertiary care hospital in Odisha. All clinical specimens were processed for bacterial identification and antimicrobial susceptibility testing using the VITEK-2 automated system (bioMérieux, Marcy-l’Étoile, France). *A. baumannii *isolates were characterized as CRAB and carbapenem-susceptible *Acinetobacter baumannii* (CSAB). CRAB susceptibility to sulbactam-durlobactam was tested by the Kirby-Bauer disc diffusion method, and results were interpreted based on the Clinical & Laboratory Standards Institute 2024 guidelines. Clinical and microbiological data were analyzed by applying appropriate statistical tests, with a p-value <0.05 considered statistically significant.

Results

Among 291 *Acinetobacter* spp. isolates, 275 (94.5%) were *A. baumannii*, and out of the 275 *A. baumannii*, 227 (82.5%) were carbapenem-resistant. Most of the CRAB-infected patients were ≥60 years old (93, 40.9%). All CRAB isolates were multidrug resistant. CRAB cases showed a significantly higher association with multiple comorbidities and risk factors, including hypertension (53.7%, 122/227), diabetes (31.1%, 72/227), malignancy (22.9%, 52/227), renal disease (33.4%, 76/227), surgery/invasive procedures/accident (33.9%, 77/227), lung disease (53.7%, 122/227), liver disease (31.2%, 71/227), bloodstream infection (57.2%, 130/227), and genitourinary infection (61.6%, 140/227) than CSAB cases. Mortality was 40% (91/227) in CRAB. The majority (98.6%, 224/227) of the CRAB isolates showed sensitivity to sulbactam-durlobactam.

Conclusions

Our study highlights the alarming rise of CRAB in a tertiary care setting in Odisha as an emerging ICU pathogen, particularly among elderly patients. The strong association with comorbidities, risk factors, and multidrug resistance underscores the need for continuous surveillance and targeted interventions. Sulbactam-durlobactam demonstrated promising activity, indicating its potential as an effective therapeutic option.

## Introduction

Antimicrobial resistance (AMR), which compromises the efficacy of antibiotics, has emerged as a silent but formidable public health concern [1]. With an estimated 4.95 million fatalities, the World Health Organization (WHO) lists AMR as one of the gravest global health crises. Major, clinically significant bacterial pathogens have strikingly high resistance levels, according to the Global Antimicrobial Resistance and Use Surveillance System (GLASS) report of 2022 [2]. AMR substantially escalates morbidity, mortality, and healthcare expenditure. It is responsible for about 700,000 deaths, which indicates a catastrophic increase to nearly 10 million deaths annually by 2050 [3].

In the hospital milieu, the AMR burden is largely driven by ESKAPE (*Enterococcus faecium*, *Staphylococcus aureus*, *Klebsiella pneumoniae*, *Acinetobacter baumannii*, *Pseudomonas aeruginosa*, and *Enterobacter* spp.) pathogens. The AMR is escalated through horizontal gene transfer, where plasmid-mediated resistance can spread rapidly within these pathogens [4]. In this scenario, *A. baumannii* has become a significant nosocomial threat globally, particularly in intensive care units (ICUs). It is intrinsically linked to a wide spectrum of healthcare-associated infections (HAIs), such as ventilator-associated pneumonia and wound, urinary tract, bloodstream, and central nervous system infections, which are caused by diverse resistance mechanisms [5].

Carbapenem resistance in *Acinetobacter baumannii* (CRAB) is particularly frightening because it thrives in hospital environments, encourages the persistence of this pathogen within healthcare facilities, and compromises conventional antimicrobial therapy [6]. Carbapenems are known as the linchpin of therapy amid broad-spectrum β-lactams, which are used for the treatment of the severe HAIs described above, especially when caused by multidrug-resistant (MDR) gram-negative organisms. MDR organisms demonstrate resistance to at least one antimicrobial agent across three or more drug classes. However, the efficacy of carbapenems is lost when resistance to last-line agents (imipenem, meropenem, doripenem, or ertapenem) develops, thereby therapeutic efficiency is critically eroded by the reduction of treatment alternatives [6,7]. This resistance is often driven by transferable carbapenemase genes that accelerate the entrenchment of multidrug resistance among bacteria [6]. The Indian Council of Medical Research (ICMR) Annual Report of the AMR Research and Surveillance Network-2024 observed evidently high carbapenem resistance (91%) in *A. baumannii* in ICU settings in India [8].

As a result, CRAB is linked with severe infections and large outbreaks in hospitalized and long-term care populations, with mortality rates of nearly 55%. This leads to the development of carbapenem resistance, which is a major clinical and infection control challenge in cases of *A. baumannii* infections [6,9]. CRAB is categorized as a “critical priority pathogen” by the Centers for Disease Control and Prevention (CDC) and WHO. It also ranks first in the WHO priority pathogen list (2018). Additionally, it continues to hold the top position on the updated Bacterial Priority Pathogen List (BPPL) 2024, emphasizing the critical need for new antimicrobial approaches [10,11]. As resistance to this class signifies widespread co-resistance and advanced antimicrobial resistance, carbapenems are commonly utilized as resistance markers [12].

CRAB epitomizes a pernicious dimension of AMR, which compounds the global AMR epidemic, undermining progress toward Sustainable Development Goal 3 (SDG), which deals with good health and well-being. It mandates healthy lives throughout the life course and is destabilized by CRAB through degradation of effective therapy and amplified risks to patient well-being [13]. The One Health paradigm has been endorsed by the WHO, which integrates human, animal, and environmental health through recognition of their deep-rooted interdependency. It ensures coordinated surveillance and control, and the framework is especially necessary, which is particularly pertinent to AMR, such as CRAB [14].

The efficacy of established agents has been compromised by the widespread misuse of antimicrobials, with the therapeutic armamentarium gradually shrinking. However, the stagnation in antibacterial innovation has broadened the treatment deficit, and the pressing need for novel strategies and equitable antibiotic access is emphasized [13]. In this context, the U.S. Food and Drug Administration in 2023 approved sulbactam-durlobactam for hospital- and ventilator-associated pneumonia due to *A. baumannii*. Durlobactam is a next-generation diazabicyclooctane β-lactamase inhibitor, which effectively restores sulbactam efficacy through potent class D inhibition. This interaction results in an approximately 32-fold reduction in sulbactam minimum inhibitory concentration (MIC_50_) and high global susceptibility rates. Clinical potency was validated in the phase III, multi-centric, *Acinetobacter* Treatment Trial Against Colistin (ATTACK) trial among patients infected with CRAB-associated pneumonia and bloodstream infection (BSI) [15,16].

Despite the fact that CRAB remains a dominant ICU-concomitant HAI pathogen, integrated clinico-microbiological data from eastern India are limited. Region-specific epidemiology, encompassing prevalence, comorbidities, and exposure-driven risk factors, profoundly shapes resistance to therapeutic outcomes. Therefore, this study characterizes CRAB isolates and evaluates the in vitro activity of sulbactam-durlobactam to generate actionable evidence for antimicrobial stewardship and infection-control interventions.

## Materials and methods

Study design and setting

This cross-sectional study was conducted at the Department of Microbiology, in association with the Department of Critical Care and Medicine of Kalinga Institute of Medical Sciences, a tertiary care teaching hospital in Bhubaneswar, located in the eastern region of Odisha, India. The study period extended for a period of six months (July 2024 to December 2024).

The clinico-microbiological profile of ICU patients, regardless of age or gender, whose clinical samples yielded *A. baumannii* on microbiological culture, was included in the study. Patients with incomplete or missing medical records, inadequate clinical or microbiological data, and duplicate isolates of a patient were excluded from the study. Data pertaining to carbapenem-susceptible *Acinetobacter baumannii *(CSAB) isolates were also collected for comparative analysis for the same study period.

Sample collection and processing

The clinical samples were received from a range of ICUs, including the Medicine, Respiratory, Neurosurgery, Neurology, Oncology, Neonatal, Pediatric, Emergency, and other specialized ICUs within the hospital. Various clinical specimens were obtained from patients, including endotracheal aspirates (ETs), blood, pus, urine, tissue biopsies, wound swabs, bronchoalveolar lavage (BAL), stool, and other body fluids. All specimens were obtained from ICU patients with suspected infections following standard aseptic techniques and arrived in the microbiology laboratory for further processing and examination.

All clinical samples were processed following standard microbiological procedures. Blood specimens were aseptically inoculated into age-specific culture bottles and incubated in an automated system (BacT/ALERT 3D; bioMérieux, Marcy-l’Étoile, France), where they were monitored for up to five days or until growth was detected. Bottles signaling positivity were then subcultured onto suitable media. General specimens were initially inoculated onto blood agar and MacConkey agar, while urine samples were cultured on cystine lactose electrolyte-deficient (CLED) agar (Hi-Media Laboratories Pvt. Ltd., Mumbai, India). The inoculated plates were then incubated aerobically at 35 ± 2°C for 18-24 hours.

Definitive species-level identification was confirmed, and antimicrobial susceptibility testing (AST) was performed using the VITEK® 2 automated identification system (bioMérieux, Marcy-l’Étoile, France). AST results, including MIC values, were obtained and interpreted according to the Clinical & Laboratory Standards Institute (CLSI) 2024 guidelines [17]. The AST panel for CRAB isolates included ampicillin-sulbactam, amikacin, ceftriaxone, ciprofloxacin, cefepime, gentamicin, levofloxacin, minocycline, piperacillin-tazobactam, ceftazidime, and trimethoprim-sulfamethoxazole. *A. baumannii* clinical isolates were classified as carbapenem-resistant, based on the resistance to one of the carbapenem agents tested: imipenem or meropenem. For uniformity in analysis, imipenem resistance was used as the defining criterion.

In vitro susceptibility of sulbactam-durlobactam

In vitro AST of sulbactam-durlobactam (10/10 µg; Hardy Diagnostics, Santa Maria, CA, USA) was performed to assess the activity of sulbactam-based agents against CRAB isolates by the Kirby-Bauer disk diffusion method. The zone of inhibition was measured at ≥17 mm as sensitive and ≤13 mm as resistant, and the isolates were categorized based on the CLSI guidelines, 2024 [17].

Data collection

All clinical specimens were received and processed in the microbiology laboratory during the study period July 2024 to December 2024). The corresponding clinical and microbiological data of ICU patients with CRAB infection were collected and thoroughly reviewed using a comprehensive data collection sheet. The dataset included both the clinical and demographic parameters, such as age, gender, underlying comorbid conditions (e.g., diabetes, hypertension, respiratory infections, urinary tract infections (UTIs), and microbiological findings. In-hospital mortality was defined as the occurrence of death during the patient’s stay in the ICU and was documented.

Statistical analysis

The collected data were analyzed, and the associations between patient characteristics, microbiological profiles, and clinical outcomes in the context of CRAB and CSAB infections were evaluated. Continuous variables were summarized as mean ± standard deviation (SD), while categorical variables were expressed as frequencies and percentages. All collected data and parameters were carefully entered into a Microsoft Excel spreadsheet. Relevant categorical tabulations, graphical illustrations, and statistical analyses were performed using Microsoft Excel (version 16.105.1), R software (version 4.5.1), and Python (version 3.9.6) for macOS as appropriate. Comparisons between categorical variables were conducted using the chi-square test, and further analyses were then performed to evaluate patterns, trends, and correlations among the collected variables. A p-value <0.05 was considered statistically significant.

Ethical clearance

Clinical samples were collected after obtaining approval from the Institutional Ethics Committee (IEC) (approval number: KIIT/KIMS/IEC/1344/2023).

## Results

**Figure 1:**
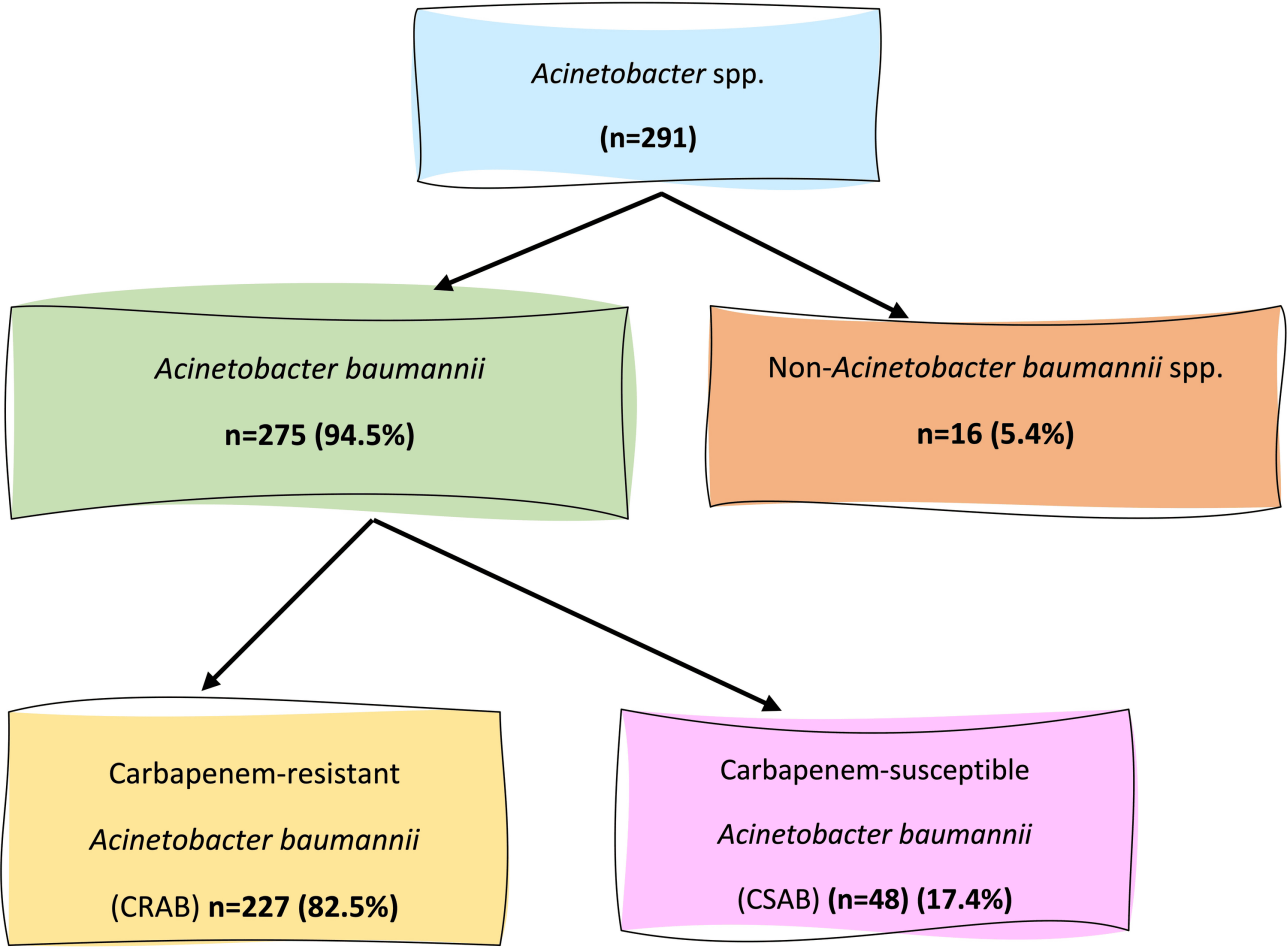
Distribution of carbapenem-resistant and carbapenem-susceptible Acinetobacter baumannii isolates.

A total of 291 *Acinetobacter* spp. isolates were recovered from clinical specimens of patients admitted to various ICUs. Of these, 275 (94.5%) were identified as *A. baumannii*, while 16 (5.4%) belonged to non-*baumannii Acinetobacter* species. Among the 275 *A. baumannii* isolates, 227 (82.5%) were found to be CRAB phenotypically, whereas 48/275 (17.4%) were CSAB. Figure 1 depicts the distribution and classification of *A. baumannii* and carbapenem-resistance status.

Among the 227 CRAB cases, males constituted the majority at 64.7% (147/227), while females accounted for 35.2% (80/227). The mean age of the patients was 52.8 ± 20.2 years, with a median age of 57 years, ranging from 6 days to 91 years, with the highest burden observed in patients aged ≥60 years (40.9%, 93/227) (Table 1).

**Table 1:** Demographic profile of ICU patients with CRAB infection (n = 227). CRAB: carbapenem-resistant *Acinetobacter baumannii*; ICU: intensive care unit

Demographic variables (n = 227)	n (%)
Gender	
Male	147 (64.7%)
Female	80 (35.2%)
Age (in years)	
<10 years	8 (3.5%)
11–20 years	7 (3.0%)
21–30 years	20 (8.8%)
31–40 years	18 (7.9%)
41–50 years	36 (15.8%)
51–60 years	45 (19.8%)
>60 years	93 (40.9%)

**Figure 2:**
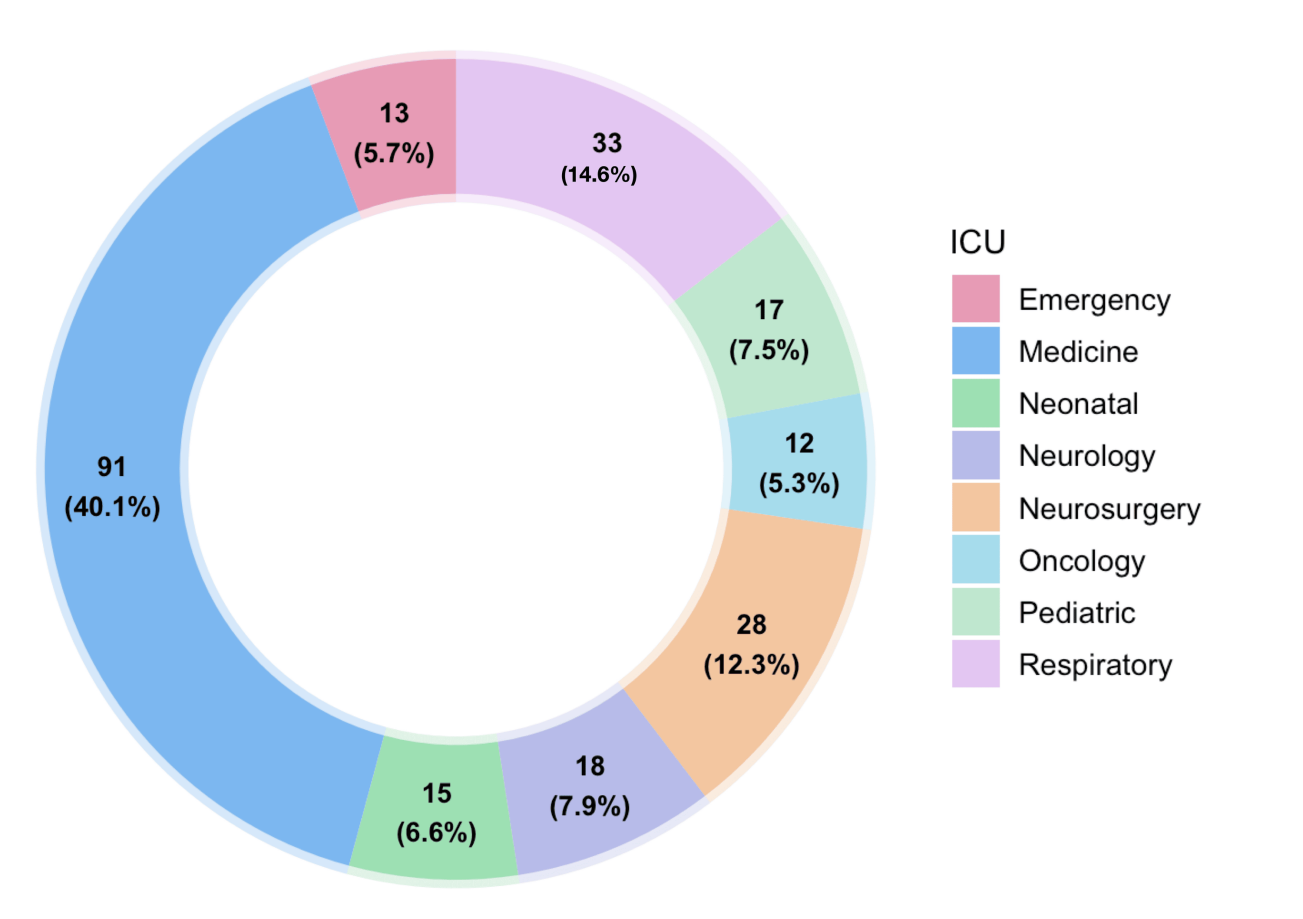
ICU-wise distribution of CRAB isolates (n = 227). Data are presented as frequency (percentage). CRAB: carbapenem-resistant *Acinetobacter baumannii*; ICU: intensive care unit

CRAB cases were most frequently reported from the Medicine ICU (40.1%, 91/227), followed by the Respiratory ICU (14.5%, 33/227), and Neurosurgery ICU (12.3%, 28/227), while CSAB isolates were consistently fewer across all ICU settings (Figure 2).

**Figure 3:**
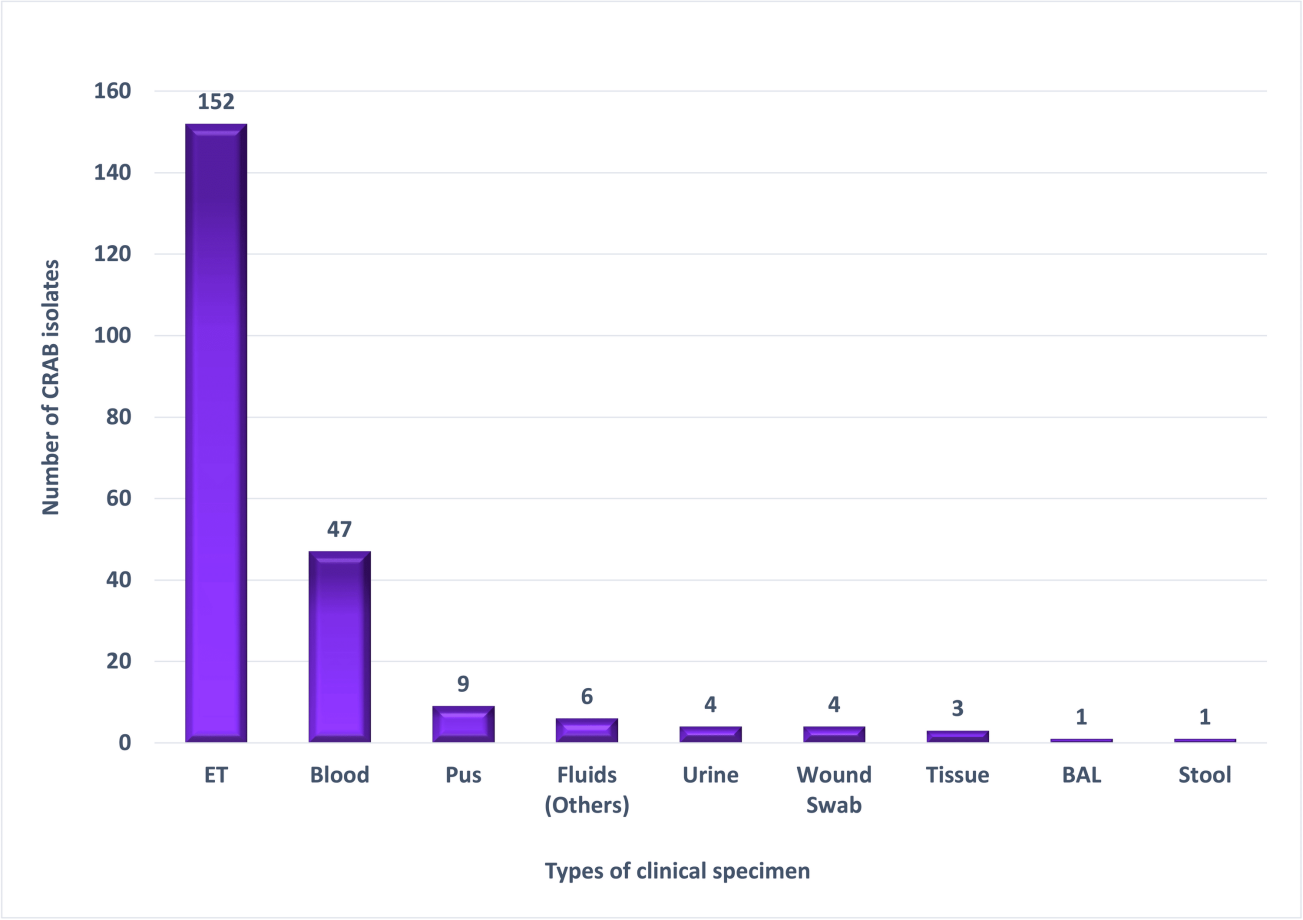
Clinical specimen-wise distribution of CRAB isolates (n = 227). Data are presented as frequency (n). CRAB: carbapenem-resistant *Acinetobacter baumannii*;* *ET: endotracheal aspirates; Fluids (others): pleural, ascitic, cerebrospinal, and synovial fluids; BAL: bronchoalveolar lavage fluid

Clinical specimens yielding CRAB showed that ET aspirates were the predominant source (67.0%, 152/227), followed by blood samples (20.7%, 47/227), while other samples contributed minimally (Figure 3).

All CRAB isolates demonstrated resistance to imipenem, which was used as the defining criterion for carbapenem resistance. The MIC values for imipenem were predominantly ≥16 µg/mL, exceeding the CLSI 2024 resistance breakpoint (≥8 µg/mL), confirming high-level phenotypic carbapenem resistance.

**Figure 4:**
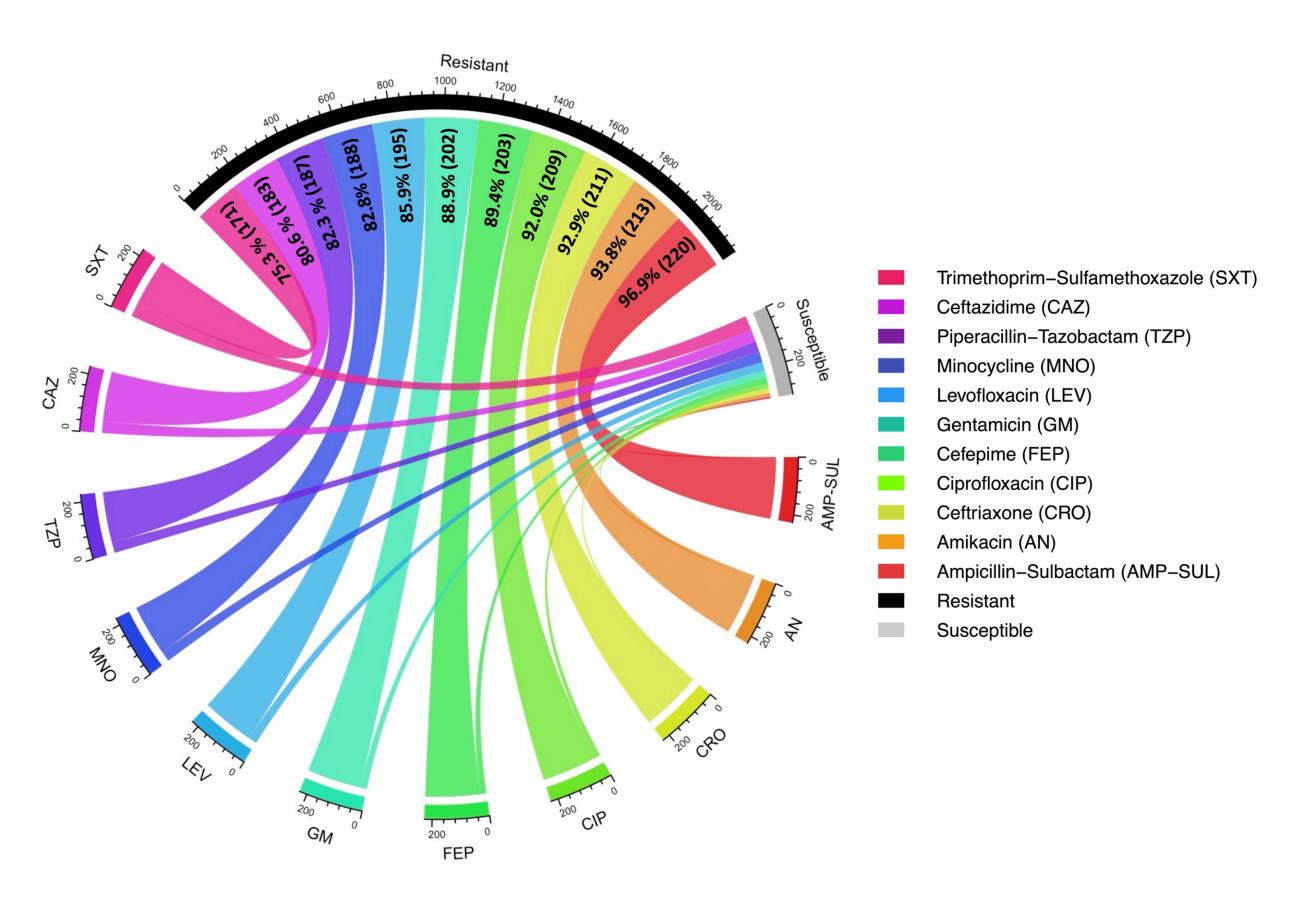
Chord diagram of the AST profile of CRAB isolates (n = 227). Resistance percentages and corresponding counts are shown in the figure. Interpretation was performed according to Clinical & Laboratory Standards Institute 2025 guidelines. AST: antimicrobial susceptibility testing; CRAB: Carbapenem-resistant *Acinetobacter baumannii*

Figure 4 depicts the AST results of CRAB isolates (n = 227) using a circular chord diagram, which includes both resistant and susceptible categories for all tested antibiotics. In this chord diagram, the outer segments represent individual antibiotics, and the chords connect each antibiotic to either the resistant or susceptible category. The thickness of each chord reflects the relative number of isolates associated with that antibiotic. Resistant segments appear wider and are connected by thicker chords, whereas susceptible segments are smaller with thinner chords. For better visualization and clarity, resistant proportions are highlighted, and percentage values are displayed on the corresponding resistant chords. The convergence of thick chords from multiple antibiotics indicates the presence of multidrug resistance among CRAB isolates. CRAB isolates exhibited markedly lower susceptibility to all of the tested antibiotics. Among all, the highest resistance was observed in ampicillin-sulbactam (96.90%, 220/227), followed by amikacin (93.8%, 213/227).

**Figure 5:**
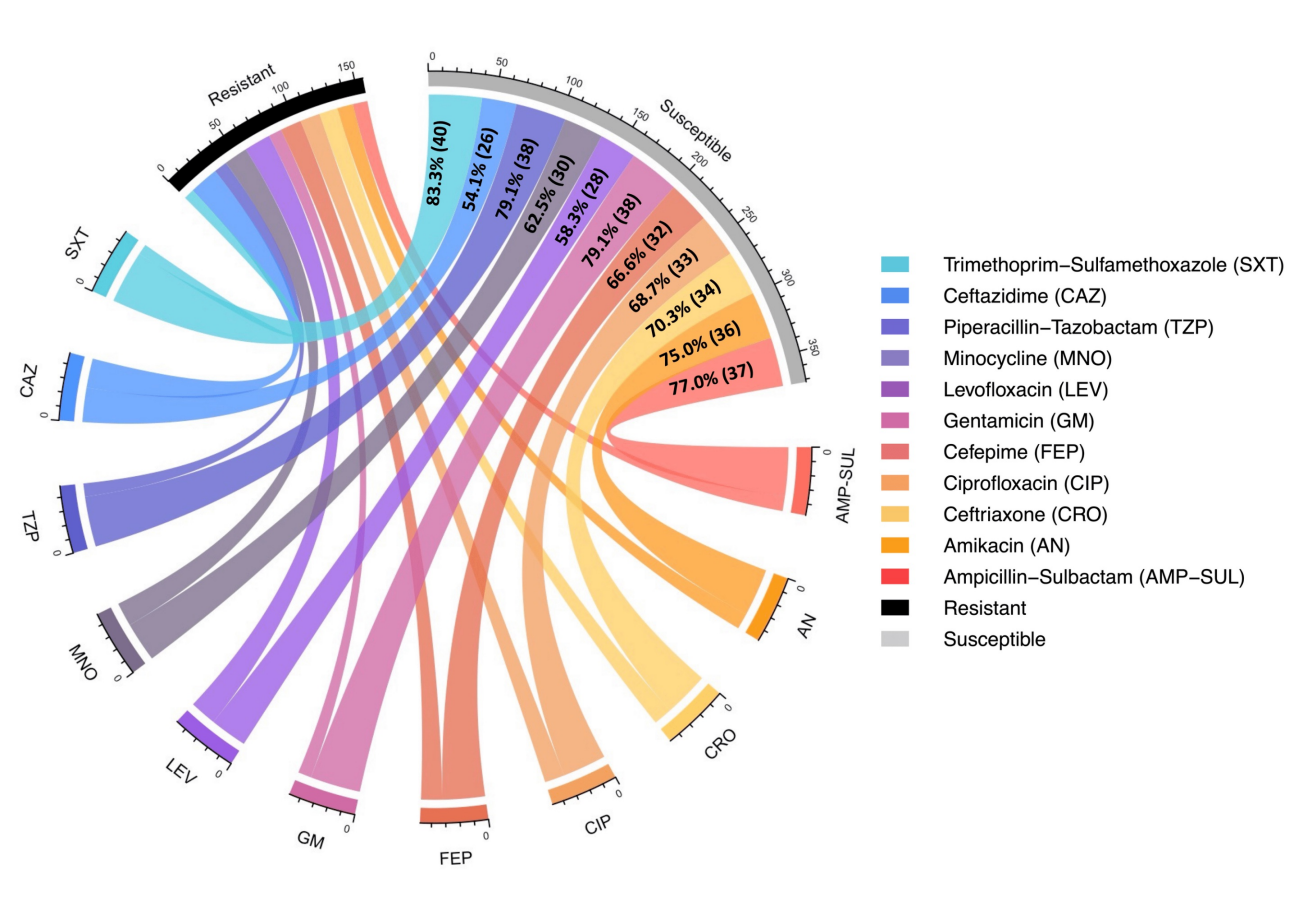
Chord diagram of the AST profile of CSAB isolates (n = 48). Susceptible percentages and corresponding counts are shown in the figure. Interpretation was performed according to Clinical & Laboratory Standards Institute 2025 guidelines. AST: antimicrobial susceptibility testing; CSAB: carbapenem-susceptible *Acinetobacter baumannii*

The chord diagram for the AST results of CSAB isolates (n = 48) is interpreted similarly (refer to Figure 4) and is shown in Figure 5. However, unlike the chord diagram for CRAB isolates, it highlights susceptible proportions of isolates. Susceptibility is represented by thicker and wider chords connecting the respective antibiotics, with percentage values displayed on the corresponding susceptible chords. This visualization allows comparison of relative susceptible contributions among CSAB isolates. Among all the antibiotics tested, CSAB isolates were mostly susceptible to trimethoprim-sulfamethoxazole (83.3%, 40/48), followed by piperacillin-tazobactam and gentamicin with the same values (79.1%, 38/48).

Resistance to all the tested antibiotics was higher among CRAB isolates. In contrast, susceptibility was higher among CSAB isolates. To evaluate this difference, a chi-square test was performed comparing resistance patterns between the two groups. All antibiotics showed statistically significant differences (p < 0.00001) in resistance patterns between CRAB and CSAB isolates.

**Figure 6:**
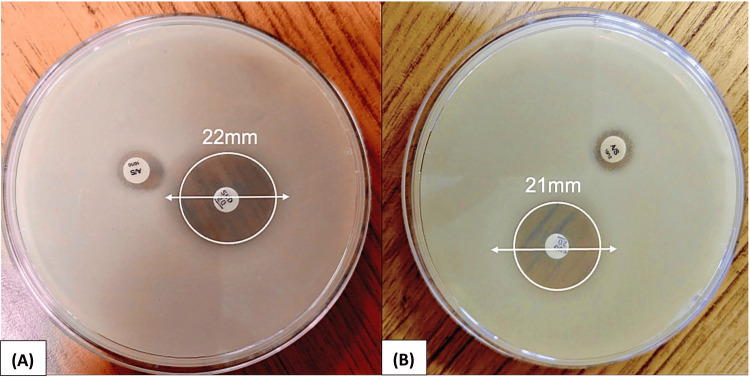
Kirby-Bauer disc diffusion for sulbactam-durlobactam against CRAB isolates (n = 227). Representative image of AST using the Kirby-Bauer disk diffusion method for sulbactam-durlobactam against CRAB isolates. (A) Zone of inhibition measuring 22 mm. (B) Zone of inhibition measuring 21 mm. AST: Antimicrobial susceptibility testing; CRAB: carbapenem-resistant *Acinetobacter baumannii*

Figure 6 illustrates a representative image of the Kirby-Bauer disc diffusion assay for sulbactam-durlobactam (10/10 µg) against CRAB isolates. The visible zone of inhibition was measured in millimeters to assess in vitro activity. The same procedure and interpretation criteria were applied uniformly to all 227 CRAB isolates included in the study, of which 98.6% (224/227) were susceptible to sulbactam-durlobactam.

CRAB infection was significantly associated with multiple comorbidities and risk factors, as summarized in Table 2. Genitourinary infection (61.6%, 140/227), followed by BSI (57.2%, 130/227), and lung disease (53.70%,122/227), were the most frequent conditions seen in CRAB cases compared with CSAB cases. Chi-square test was applied for statistical analysis of the variables for comparison between CRAB and CSAB-infected patients.

**Table 2:** Comparison of comorbidities and risk factors between CRAB (n = 227) and CSAB (n = 48) infected patients. CRAB: carbapenem-resistant *Acinetobacter baumannii*;* *CSAB: carbapenem-susceptible *Acinetobacter baumannii*; BSI: bloodstream Infection; UTI: urinary tract infection; χ²: chi-square test; df: degrees of freedom

Baseline comorbidities/risk factors	CRAB (n = 227) (%)	CSAB (n = 48) (%)	χ² value	df	P-values
UTI/Prostate/Vaginal infection (148)	140 (61.6%)	8 (16.6%)	32.292	1	<0.001
Surgery/Invasive procedure/Accident (83)	77 (33.9%)	6 (12.5%)	8.627	1	0.003
Renal disease (83)	76 (33.4%)	7 (14.5%)	6.714	1	0.010
Organ transplant (53)	46 (20.2%)	7 (14.5%)	0.821	1	0.365
Malignancy (56)	52 (22.9%)	4 (8.3%)	5.189	1	0.023
Lung disease/Pneumonia (133)	122 (53.7%)	11 (22.9%)	15.078	1	< 0.001
Liver disease (78)	71 (31.2%)	7 (14.5%)	5.434	1	0.020
Hypertension (132)	122 (53.7%)	10 (20.8%)	17.193	1	<0.001
Diabetes (78)	72 (31.1%)	6 (12.5%)	7.202	1	0.007
Cardiac disease (59)	51 (22.4%)	8 (16.6%)	0.791	1	0.374
BSI/Sepsis/Septicemia (144)	130 (57.2%)	14 (29.1%)	12.544	1	<0.001
Brain injury/Meningitis (126)	104 (45.8%)	22 (45.8%)	5.377	1	0.998

**Figure 7:**
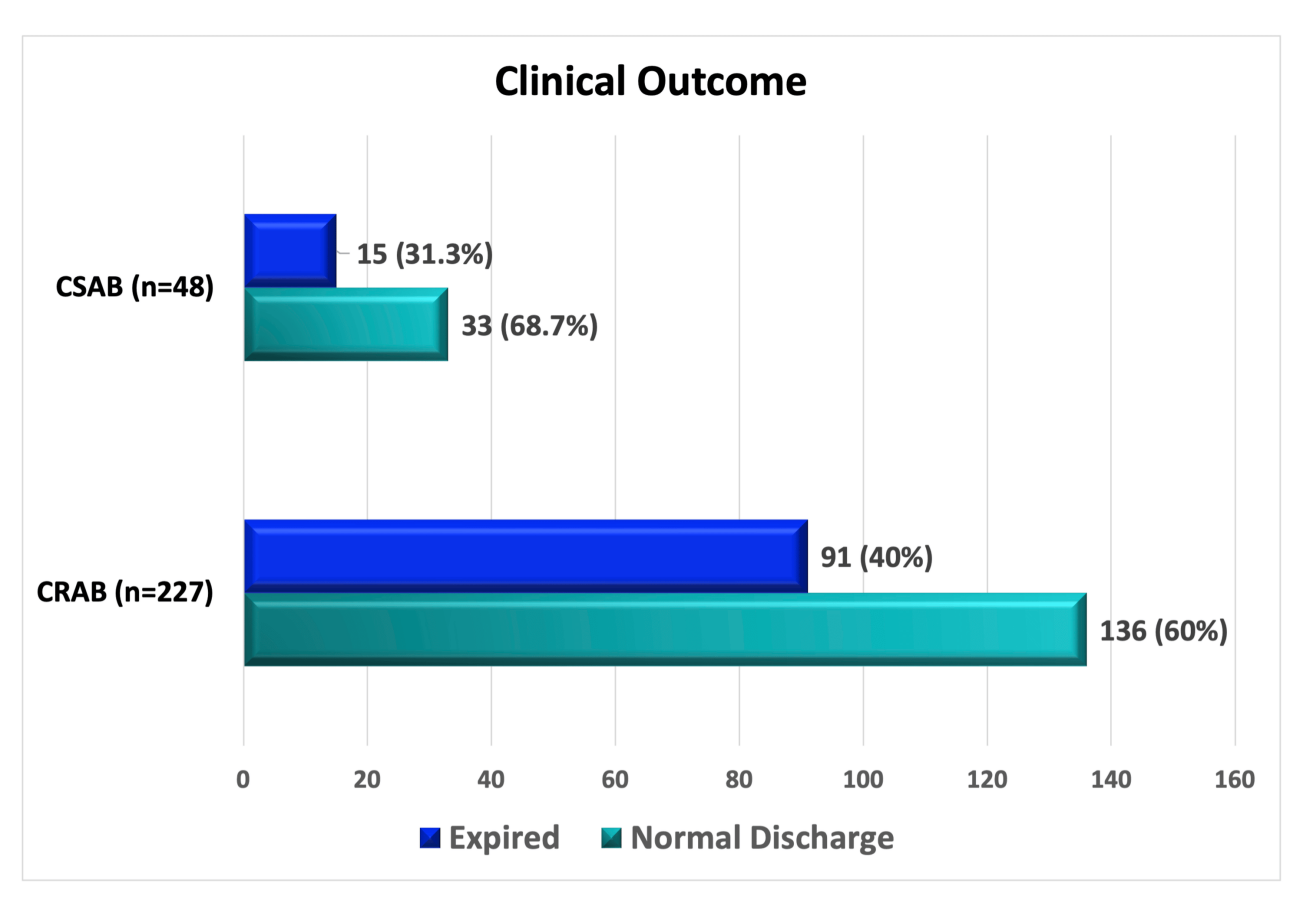
Clinical outcome among CRAB (n = 227) and CSAB (n = 48) infected patients. Data are presented as frequency (percentage). CRAB: carbapenem-resistant *Acinetobacter baumannii*; CSAB: carbapenem-susceptible *Acinetobacter baumannii*

Overall, in-hospital mortality among CRAB-infected patients was 40% (91/227). However, there was no statistically significant association (p = 0.52) between CRAB infection and mortality rate (Figure 7).

## Discussion

Infections caused by drug-resistant organisms are increasingly reported in hospitals, particularly in ICUs, where critically ill patients are extremely vulnerable due to impaired immunity and extensive use of invasive devices. The widespread use of carbapenems has led to a significant rise in carbapenem resistance, especially among non-fermenting Gram-negative bacilli (NFGNB). Among these, CRAB has emerged as a general cause of HAIs in ICUs, causing severe infections such as pneumonia, BSIs, and UTIs. The persistence of CRAB in the hospital environment and its limited treatment options make it a critical threat to patient outcomes in ICU settings [18]. This study of CRAB in our ICU setting from a tertiary care hospital in eastern India provides insight into the burden and characteristics of CRAB against the backdrop of widespread carbapenem resistance among NFGNB.

In our setting, a definite male predominance was observed (64.7%, 147/227) than females (35.2%, 80/227), and the mean age was 52.81 years, with the majority of infections occurring in patients older than 60 years (40.9%, 93/227). These findings closely parallel the findings of Chen et al., who found a mean age of 66.6 years and a male preponderance of about 66% in patients with CRAB infection in their ICU settings [19].

A substantial local prevalence of carbapenem resistance was indicated by the fact that around 82.5% (227/291) of the *A. baumannii* isolates from samples obtained from ICU patients were CRAB. Similar incidence has been documented from tertiary-care hospitals in India, where CRAB rates usually vary from approximately 40% to 75%, which reflects regional variation and differences in patient populations [20]. National surveillance data from the ICMR AMRSN, 2024, further corroborate our findings, reporting approximately 91% CRAB, further supporting the endemic nature of CRAB in Indian ICUs [8]. A recent study from Jordan reported 96.7% CRAB in their ICU settings (Ababneh et al.) [21]. Carbapenem resistance among *A. baumannii* varies widely across Europe, ranging up to 96% across EU/EEA countries, with ≥50% resistance reported in Southern and Southeastern Europe, supporting that high CRAB prevalence is not unique to India (Boutzoukas et al., JAC-AMR, 2025) [22].

The similarity in such high rates of CRAB across different regions may reflect common shared challenges, including gaps in antimicrobial stewardship, inadequate infection-control interventions, and the need for changes in ICU policies, which collectively contribute to the sustained global burden.

CRAB recovery in our ICU was skewed toward the Medicine ICU (40.1%, 91/227), followed by the Respiratory ICU (14.5%, 33/227). Sample-wise, ET aspirates dominated at 67% (152/227), followed by blood samples at 20.7% (47/227). A comparable observation was seen in the study reported by Banerjee et al., where CRAB was identified as the leading pathogen in hospital-acquired pneumonia and a major contributor to BSIs, reflecting its affinity for ventilated hosts that supports the pattern we observed [23].

Moreover, CRAB isolates demonstrated markedly higher resistance than CSAB isolates across all commonly tested antibiotic classes. Elevated resistance was observed in ceftriaxone (92.9%, 211/227 vs. 29.1%, 14/48), cefepime (89.4%, 203/227 vs. 33.3%, 16/48), ciprofloxacin (92%, 209/227 vs. 31.2% 15/48), amikacin (93.8%, 213/227 vs. 25%, 12/48), gentamicin (88.9%, 202/227 vs. 20.8%, 10/48), and piperacillin-tazobactam (82.3%, 187/227 vs. 20.8%, 10/48). The results were significantly higher among CRAB isolates (p < 0.00001 for all agents) than CSAB isolates, which suggests that carbapenem resistance is a noticeable MDR phenotype. This resistance profile is strongly supported by ICMR-AMRSN, 2024 ICU data, which reported very low susceptibility among *A. baumannii* isolates to the same agents: cefepime (4%), ceftazidime (5.7%), piperacillin-tazobactam (6.7%), and amikacin (9.4%) [8]. Similar resistance magnitudes have been documented in an Indian tertiary care study by Banerjee et al., where CRAB isolates showed resistance rates ranging from 76.99% to 92.01%, closely mirroring the resistance spectrum observed in our findings, translating to resistance levels exceeding 90% in ICU settings [23]. Ampicillin-sulbactam showed poor activity against CRAB isolates in our study, as 96.9% (220/227) of isolates showed resistance, consistent with the reports describing reduced effectiveness of sulbactam-based regimens in CRAB [5,12]. This can robustly validate our findings and confirm that CRAB in ICU settings is consistently associated with extreme multidrug resistance, leaving few effective conventional therapeutic options.

Additionally, 98.6% (224/227) of CRAB isolates were susceptible to sulbactam-durlobactam by the disk diffusion method, indicating preserved in vitro activity. Indian clinical data remain limited, but international surveillance studies have demonstrated consistently high in vitro activity of sulbactam-durlobactam, with inhibition of 96.9% of CRAB isolates in global collections [24]. Although sulbactam-durlobactam demonstrated promising in vitro activity against CRAB isolates, its translation into routine clinical practice warrants a well-defined implementation strategy. Its administration must be aligned with antimicrobial stewardship principles and ideally reserved for confirmed or high-risk CRAB infections to preserve its efficacy. In low- and middle-income countries (LMICs), considerations such as cost-effectiveness, drug availability, and access may influence its incorporation in ICU settings.

CRAB infection in our ICU setting was strongly associated with a higher burden of underlying comorbidities compared to CSAB infection. Hypertension (53.7%, 122/227), diabetes (31.1%, 72/227), renal disease (33.4%, 76/227), malignancy (22.9%, 52/227), liver disease (31.2%, 71/227), prior surgery/invasive procedures (33.9%, 77/227), lung disease/pneumonia (53.7%, 122/227), BSI (57.2%, 130/227), and genitourinary infections (61.6%, 140/227) were all significantly more frequent among CRAB patients. This highlights the convergence of chronic illness, severe infection, and healthcare exposure in this population. Similar risk profiles for CRAB patients were consistently reported from multinational ICU studies, where metabolic disorders, organ dysfunction, malignancy, and respiratory disease predominate among affected patients [9].

The invasive nature of CRAB and its prediction for critically ill patients with severe infections are revealed by the strong association with pneumonia and BSI, particularly in ICU settings. This pattern is aligned with evidence from ICU-based studies published in India and international settings, where CRAB mainly causes BSI and severe pneumonia [25,26].

The 40% (91/227) mortality rate among CRAB-infected patients in our study emphasizes the serious clinical consequences of these infections in ICU patients. According to the report, the death rate for ICU patients ranges from 45% to 60%, and when these organisms have ubiquitous drug resistance, it can reach 80% [27].

However, this study has certain limitations. First, as it was conducted at a single tertiary care center, it may limit the generalizability of the findings to other healthcare settings. In addition, susceptibility testing for sulbactam-durlobactam was performed using the disk diffusion method, as broth microdilution was not performed. Therefore, the findings reflect in vitro susceptibility patterns and not clinical efficacy, and should be interpreted within this methodological context. The absence of detailed data on ICU stay duration, prior antibiotic exposure, and device-related factors (such as mechanical ventilation and catheterization) restricted a more comprehensive assessment of risk factors. Additionally, molecular characterization of resistance mechanisms was not performed, which could have provided deeper insights into the genetic basis of carbapenem resistance. Finally, clinical outcomes beyond in-hospital mortality were not evaluated, limiting the assessment of long-term patient outcomes.

## Conclusions

This study shows that the growth of CRAB* *in the ICU of a tertiary care setting in Odisha, with a notable predilection for elderly patients, is alarming. The important need for ongoing surveillance and focused infection-control measures in critical care settings is highlighted by the substantial correlation between CRAB infection and numerous comorbidities, clinical risk factors, and widespread AMR. Sulbactam-durlobactam’s encouraging in vitro efficacy against CRAB isolates points to its potential role as an effective therapeutic option in the management of these challenging infections. These findings further emphasize the need for strengthened antimicrobial stewardship programs, optimized ICU antibiotic policies, and sustained infection-control interventions to curb the ongoing burden of CRAB and AMR.
